# Orthohantavirus Pathogenesis and Cell Tropism

**DOI:** 10.3389/fcimb.2020.00399

**Published:** 2020-08-04

**Authors:** Danny Noack, Marco Goeijenbier, Chantal B. E. M. Reusken, Marion P. G. Koopmans, Barry H. G. Rockx

**Affiliations:** ^1^Department of Viroscience, Erasmus University Medical Center, Rotterdam, Netherlands; ^2^Department of Internal Medicine, Erasmus University Medical Center, Rotterdam, Netherlands; ^3^Center for Infectious Disease Control, National Institute for Public Health and the Environment, Bilthoven, Netherlands

**Keywords:** orthohantavirus, hantavirus, hemorrhagic fever with renal syndrome, hantavirus cardiopulmonary syndrome, tropism, endothelium, pathogenesis

## Abstract

Orthohantaviruses are zoonotic viruses that are naturally maintained by persistent infection in specific reservoir species. Although these viruses mainly circulate among rodents worldwide, spill-over infection to humans occurs. Orthohantavirus infection in humans can result in two distinct clinical outcomes: hemorrhagic fever with renal syndrome (HFRS) and hantavirus cardiopulmonary syndrome (HCPS). While both syndromes develop following respiratory transmission and are associated with multi-organ failure and high mortality rates, little is known about the mechanisms that result in these distinct clinical outcomes. Therefore, it is important to identify which cell types and tissues play a role in the differential development of pathogenesis in humans. Here, we review current knowledge on cell tropism and its role in pathogenesis during orthohantavirus infection in humans and reservoir rodents. Orthohantaviruses predominantly infect microvascular endothelial cells (ECs) of a variety of organs (lungs, heart, kidney, liver, and spleen) in humans. However, in this review we demonstrate that other cell types (e.g., macrophages, dendritic cells, and tubular epithelium) are infected as well and may play a role in the early steps in pathogenesis. A key driver for pathogenesis is increased vascular permeability, which can be direct effect of viral infection in ECs or result of an imbalanced immune response in an attempt to clear the virus. Future studies should focus on the role of identifying how infection of organ-specific endothelial cells as well as other cell types contribute to pathogenesis.

## Introduction

The genus of orthohantaviruses in the family of *Hantaviridae* comprises emerging zoonotic negative-sense RNA viruses belonging to the recently reclassified order of *Bunyavirales* (Abudurexiti et al., 2019). Orthohantavirus strains are closely associated with specific rodent species or insectivores, as their natural reservoir hosts (Plyusnin and Morzunov, 2001; Zhang, 2014). Orthohantaviruses generally cause asymptomatic persistent infections in their reservoirs, and transmission between reservoir species primarily occurs via aerosolized urine, although wounding may also play a role in rodent-to-rodent transmission due to the presence of infectious virus in saliva (Glass et al., 1988; Kariwa et al., 1998). Some orthohantaviruses are capable of causing disease in humans following inhalation of aerosolized excreta from infected rodents (Lee and Johnson, 1982). Humans are considered dead-end hosts as they generally do not spread infectious virus efficiently. Although limited person-to-person transmission has been reported for Andes orthohantavirus (ANDV) (Padula et al., 1998; Martinez-Valdebenito et al., 2014). According to estimations, more than 20,000 annual cases of orthohantavirus-related disease occur worldwide with case fatality rates up to 40% (Schmaljohn and Hjelle, 1997; Alonso et al., 2019). To date, no United States Food and Drug Administration (FDA)–or European Medicines Agency (EMA)—approved specific treatments or vaccination strategies exist.

Orthohantaviruses can be divided into Old- and New World viruses due to the geographic distribution of their reservoir species with the exception of the worldwide presence of an Old World rodent: the wild rat. Currently, over 50 species of orthohantaviruses are known of which at least 24 are able to cause disease in humans (Jonsson et al., 2010; Reusken and Heyman, 2013; de Oliveira et al., 2014; Jiang et al., 2017). Clinical outcomes and disease severity in human cases largely depend on the virus species. Following respiratory transmission, orthohantaviruses can cause two distinct clinical outcomes, depending on the virus strain: hemorrhagic fever with renal syndrome (HFRS) or hantavirus cardiopulmonary syndrome (HCPS) (Hjelle and Torres-Perez, 2010; Jonsson et al., 2010).

Orthohantavirus pathogenesis is complex and the exact pathological mechanisms remain unknown. In general, pathogenesis seems to be associated with dysregulation of hemostasis, immune responses, and vascular permeability during infection due to infection of endothelial cells (ECs) lining the walls of blood vessels (Jonsson et al., 2010; Mackow et al., 2014). Immunopathology likely plays an important role in the development of disease (Rasmuson et al., 2016). In rodent reservoir hosts pro-inflammatory and antiviral immune responses are locally suppressed (e.g., by regulatory T cell responses) to establish viral persistence, without developing disease (Easterbrook et al., 2007; Schountz et al., 2007; Easterbrook and Klein, 2008a), suggesting that a lack of such regulation in humans may result in disease.

Studies into the pathogenesis of orthohantaviruses in humans have been hampered by the limited availability of clinical samples. Patient samples from the acute phase are frequently unavailable as incubation periods can take up several weeks before patients display clinical symptoms and orthohantavirus infection is often underdiagnosed (Goeijenbier et al., 2014; Sane et al., 2014). Therefore, animal models to study experimental infections are crucial in understanding the early steps in the pathogenesis of HFRS and HCPS. Unfortunately, development of laboratory animal models which recapitulate the clinical presentation of human infections has proven to be challenging. There are a very limited number of animal models to study orthohantavirus-induced disease, as reviewed in Golden et al. (2015). To date, the best characterized experimental infection model of *nephropathia epidemica* (NE; a mild form of HFRS) is Puumala orthohantavirus (PUUV) infection in non-human primates (NHP) exhibiting renal symptoms including transient proteinuria and microhematuria together with viral antigen distribution similar to that seen in human cases (Groen et al., 1995), while experimental *in vivo* models for HFRS remain largely unsuccessful in recapitulating the disease seen in humans (Golden et al., 2015). The best characterized HCPS disease models are ANDV infection in Syrian hamsters, and ANDV and Sin Nombre orthohantavirus (SNV) infection in NHP, which all recapitulate human disease (Hooper et al., 2001; Wahl-Jensen et al., 2007; Safronetz et al., 2011).

Multiple factors can determine outcome of orthohantavirus infection, such as the ability of pathogenic (i.e., associated with clinical symptoms in humans) orthohantaviruses to inhibit antiviral responses whereas non-pathogenic viruses elicit innate responses that limit viral replication in humans (Geimonen et al., 2002; Kraus et al., 2004). Additionally, differences in receptor usage are believed to be one of the crucial determinants of pathogenicity (Gavrilovskaya et al., 2002). Specific integrins (α_v_β_3_) are widely reported as receptors through which both HFRS and HCPS orthohantaviruses can enter host cells *in vitro* (Gavrilovskaya et al., 1998, 1999; Larson et al., 2005; Bondu et al., 2017). Recently, additional proteins like protocadherin-1 (Jangra et al., 2018), decay-accelerating factor/CD55 (Krautkramer and Zeier, 2008), and the receptor for the globular head domain of complement C1q/p32/p33 (Choi et al., 2008) have been described as (co-)receptors for cell entry *in vitro*. However, their cell and tissue distribution does not explain the differences in clinical outcome between HFRS and HCPS viruses (Avraamides et al., 2008; Gavrilovskaya et al., 2010; Teoh et al., 2016).

The aim of this review is to provide an updated overview of the cell and tissue tropism of pathogenic orthohantaviruses and discuss how infection of these cell types could lead to pathogenesis based on *in vivo* data and supplemented with *in vitro* data. In addition to the role of endothelium in pathogenesis, we also focus on other cells potentially targeted in five key organ systems that are most frequently studied in the context of orthohantavirus infection and pathogenesis, e.g., lung, heart, kidney, liver, and spleen. We hypothesize that cells other than ECs play an important role in the pathogenesis of orthohantaviruses and the development of distinct clinical syndromes. We review differences in cell tropism between viruses with different clinical outcomes (HFRS and HCPS), as well as differences between human and reservoir hosts to provide novel hypotheses on virus and host-specific pathways involved in disease in humans. This is crucial for identifying novel potential therapeutic targets.

## Distinct Clinical Outcomes of Orthohantavirus Infection

HFRS is typically characterized by fever, thrombocytopenia, and acute kidney injury. In severe cases internal hemorrhaging caused by increased vascular permeability can even occur (Schmaljohn and Hjelle, 1997; Jonsson et al., 2010; Vaheri et al., 2013). Hantaan (HTNV), Seoul (SEOV), and Dobrava-Belgrade (DOBV) orthohantaviruses are mainly associated with severe presentation of HFRS with mortality rates of 5–15% (Papa, 2012; Hepojoki et al., 2014). PUUV is the most prevalent orthohantavirus circulating in Europe and Russia causing NE with thousands of cases each year and a mortality rate of <0.1% (Krautkramer et al., 2013; Tkachenko et al., 2019). NE patients suffer from less severe kidney complications and less often hypotension, thrombocytopenia, and hematuria compared to HFRS cases (Jonsson et al., 2010). Tula (TULV) orthohantavirus infections have only been described in patients with severe comorbidities often related to immune suppression (Zelena et al., 2013). HCPS is a severe acute disease which mainly affects the lungs. Early non-specific flu-like symptoms rapidly develop to pulmonary edema, hypotension, and shock (Hallin et al., 1996; Khan et al., 1996; Macneil et al., 2011). ANDV and SNV are responsible for causing the majority of HCPS cases with fatality case rates up to 40% (Jonsson et al., 2010). More recently, it is becoming increasingly clear that the clinical differences between HFRS and HCPS are less distinct, with more frequent detections of respiratory disease in HFRS patients (Clement et al., 1994, 2014; Schutt et al., 2004; Gizzi et al., 2013) and kidney involvement in HCPS patients (Passaro et al., 2001; Peters and Khan, 2002). Thrombocytopenia (Connolly-Andersen et al., 2015; Latus et al., 2015) and vascular leakage (Gorbunova et al., 2010; Connolly-Andersen et al., 2015) are direct indicators for disease severity in both HFRS and HCPS.

## Pathological Outcomes Following Endothelium Infection

### Infection of Human Endothelium

**Figure 1:**
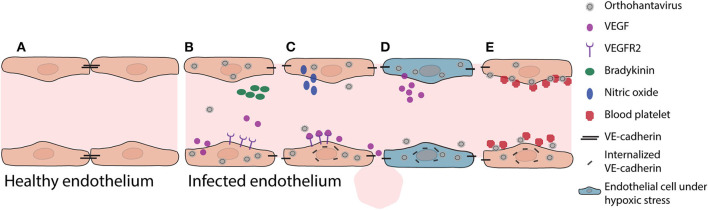
Pathogenic mechanisms in vascular endothelium during initial orthohantavirus infection. **(A)** Healthy vascular ECs contain a tightly regulated barrier, mainly based on adherens junction molecules such as VE-cadherin. **(B)** Important soluble factors that maintain this barrier function are bradykinin and VEGF. As response to infection, ECs produce and secrete VEGF. **(C)** Local VEGF binds to endothelial receptors and disengages adherens junctions by increased nitric oxide production and internalization of VE-cadherin. **(D)** Under hypoxic conditions (for instance due to pulmonary edema), these effects are even expanded as VEGF production is increased, causing increased vascular permeability. **(E)** In addition, orthohantavirus particles present on the endothelial cell surface recruit quiescent platelets to endothelial cell surfaces. This increased consumption of blood platelets may contribute in part to thrombocytopenia. Both the permeabilizing effects of secreted VEGF and the recruitment of platelets lead to internalization of VE-cadherin (i.e., loss of endothelial barrier function).

ECs are highly specialized cells which line the interior wall of blood and lymphatic vessels. ECs vary in phenotypical features and function, between different organs, including differences in expression of adhesion molecules and secretion products. ECs play an important role in vascular permeability, platelet activation, coagulation, and immune responses (Figure 1). Following entry via the respiratory tract through a yet unknown mechanism, orthohantaviruses infect ECs (primarily microvascular ECs) and subsequently spread to infect EC in almost all major organs in humans. Infection of ECs generally does not cause a cytopathic effect, but instead can lead to extensive impairment of EC functions, including barrier integrity, adhesion factors, and fluid clearance from tissues by lymphatic vessels and capillary tone regulation (Dalrymple and Mackow, 2014; Mackow et al., 2014). As a result, infection of microvascular EC barrier functions can lead to capillary leakage, a key mechanism of pathogenesis during HFRS/NE and HCPS (Yanagihara and Silverman, 1990; Duchin et al., 1994; Zaki et al., 1995; Geimonen et al., 2002). Interestingly, while orthohantaviruses infect ECs in most major organs, organ dysfunction is only reported in specific organs and depends on the causative virus. HFRS viruses generally infect the microvasculature of the kidneys, specifically targeting glomerular and tubular ECs (Hung et al., 1992; Kim et al., 1993; Groen et al., 1996; Krautkramer et al., 2011, 2013). Pulmonary and splenic microvascular beds have also been reported as targets for infection (Hautala et al., 2002; Rasmuson et al., 2011; Clement et al., 2014; Sironen et al., 2017). HCPS viruses mainly target the pulmonary microvasculature (Zaki et al., 1995; Green et al., 1998; Toro et al., 1998). Additionally, these viruses can infect microvessels in the heart, kidneys, liver, and spleen (Nolte et al., 1995; Zaki et al., 1995; Green et al., 1998; Toro et al., 1998; Saggioro et al., 2007). The mechanisms of distinct organ-specific dysfunction during HFRS and HCPS remain largely unknown. ECs from different large and microvascular vessels from different organs are considered phenotypically distinct with correspondingly characteristic gene expression profiles (Swerlick and Lawley, 1993; Chi et al., 2003). Furthermore, the microvessel wall has a more intimate association with the extracellular matrix compared to larger blood vessels, which might facilitate viral spread to other tissues.

### Pathogenesis

Increased vascular permeability by infected microvasculature is a central feature of pathogenesis leading to HCPS and HFRS. For instance, during HCPS (and to a lesser extent HFRS) increased permeability can lead to pulmonary edema, which causes severe problems such as oxygenation and ventilation problems. Oxygenation problems, leading to hypoxia, modulates actin cytoskeleton, and contractile proteins leading to further increased permeability (An et al., 2005). Hypoxic conditions also result in elevated vascular endothelial growth factor (VEGF) levels in pulmonary edema fluids (Figure 1) (Gavrilovskaya et al., 2012, 2013). Secreted VEGF binds to receptors (e.g., vascular endothelial growth factor receptor 2; VEGFR2) on ECs, acting locally to disassemble adherens junctions and induce elevated endothelial cell permeability (Dvorak, 2010; Gavard, 2014). The *in vitro* identified orthohantavirus entry receptor integrin α_v_β_3_ is vital for regulating VEGF by forming complexes with VEGFR2, which are important for multiple cellular activities such as migration, survival, and angiogenesis (Robinson et al., 2004; Gavrilovskaya et al., 2008; Somanath et al., 2009; Dvorak, 2010). During initial orthohantavirus infection (Figure 1) localized increases of VEGF contribute to pathogenesis through enhanced endothelial permeability by causing higher production of nitric oxide (NO), internalization of VE-cadherin, and possibly redistribution of zonula occludens-1 (ZO-1) in renal cells (Groeneveld et al., 1995; Klingstrom et al., 2002; Gorbunova et al., 2011; Krautkramer et al., 2011; Dalrymple and Mackow, 2014). Sustained systemic elevations of VEGF may contribute to endothelial repair and convalescence later in infection. Different inhibitors involved in VEGF signaling are able to decrease orthohantavirus-induced permeability increases both *in vitro* and *in vivo* (Gorbunova et al., 2011; Bird et al., 2016). In addition to VEGF, bradykinin is an important mediator of vascular permeability (Liu et al., 2008; Kottke and Walters, 2016). Limited evidence suggests a role of bradykinin in orthohantavirus pathogenesis (Antonen et al., 2013; Taylor et al., 2013).

A second pathological event during early phase of infection is that orthohantavirus infection can result in coagulation abnormalities. Orthohantavirus particles cluster on the surface of ECs (e.g., pulmonary microvascular beds) (Goldsmith et al., 1995; Gavrilovskaya et al., 2010) and this accumulation recruits quiescent platelets to ECs (Figure 1) (Gavrilovskaya et al., 2010). This β_3_ integrin-dependent platelet consumption may play a role in development of the observed acute thrombocytopenia, since it would result in wasting or loss of platelets adhered to infected ECs (Gavrilovskaya et al., 2010; Goeijenbier et al., 2015). This can also cause an increase of VEGFR2 phosphorylation and internalization of VE-cadherin from adherens junctions contributing to barrier function impairment and edema (Gavrilovskaya et al., 2002, 2010; Dehler et al., 2006; Bates, 2010; Gorbunova et al., 2011; Dalrymple and Mackow, 2014). In addition, disseminated intravascular coagulation without signs of hemorrhaging, major thrombosis or damage to the vascular ECs can be observed during the terminal stage of patients infected with SNV (Nolte et al., 1995; Zaki et al., 1995). These could also result in major decreases of clotting factors and platelet levels, promoting vascular leakage and hemorrhaging.

A third aspect of pathogenesis is not only described as virus-induced EC dysfunction but rather the result of immune-modulated effects (Temonen et al., 1996; Mori et al., 1999; Khaiboullina et al., 2017). There are two local immunopathological mechanisms that could contribute to the pathogenesis observed during HFRS and HCPS (Terajima and Ennis, 2011). First, early antiviral and inflammatory responses aid in eliminating virus, thereby concurrently impairing EC function by secreting large amounts of cytokines, such as tumor necrosis factor alpha (TNF-α) and interleukin-6 (IL-6) (Mori et al., 1999; Maes et al., 2006). Second, if these responses are insufficient and virus clearance is delayed, prolonged inflammation can alter EC function and cause disruption of fluid barriers (Gavrilovskaya et al., 2008; Hammerbeck and Hooper, 2011).

Finally, damaged or detached ECs can be replaced by migration of adjacent ECs or mobilization of circulating endothelial progenitor cells (Sabatier et al., 2009). Recovery of symptoms due to orthohantavirus infection has been linked to appearance of high levels circulating endothelial progenitor cells (Krautkramer et al., 2014). However, whether circulating endothelial progenitor cells initiate disease recovery or are involved in the spread and pathogenesis requires further investigation.

### Distinct Immune Responses to Infection in Non-diseased Reservoir

In reservoir rodents, orthohantaviruses are also primarily endotheliotropic (Netski et al., 1999; Maas et al., 2019). However, very little is known about the effect of orthohantavirus infection on the function and host responses by these cells. Instead, most studies have focused on the differential immunological responses that occur in reservoir rodents preventing them from developing disease. Studies on SEOV demonstrate that infection causes increases of immunoregulatory factors (e.g., expression of *Foxp3* and *Tgf* β) in pulmonary ECs and alveolar macrophages, respectively (Li and Klein, 2012). This contributes to a shift in CD4+T cell differentiation toward a more regulatory T cell phenotype during infection (Easterbrook and Klein, 2008a; Li and Klein, 2012). These data suggest that this local immunological shift may prevent complete viral clearance, hence causing persistence, as reviewed in Easterbrook and Klein (2008a). In addition, these data suggest that the pathogenesis of orthohantaviruses is at least in part the result of immunopathological responses that are controlled in reservoir species but not in humans.

## HFRS/NE in Humans and Disease Models

### Conducting Airways

While ECs are an important target for orthohantavirus infection, other cells likely play a role in entry and pathogenesis. Since cells of the conducting airways are the first to come into contact with orthohantavirus particles upon inhalation, identifying which cells are initially infected is of particular interest. To date, no data are available on the ability of HFRS-associated orthohantaviruses to target respiratory epithelial cells of the conducting airways.

### Lungs

Nevertheless, pulmonary involvement during HFRS has been reported in PUUV-infected patients with NE, in which virus-infected cells can be detected in bronchoalveolar lavage fluids (Rasmuson et al., 2016). Although pulmonary involvement during HFRS/NE is not considered a common clinical sign, post-mortem findings in severe NE cases have demonstrated extensive interstitial edema and mononuclear cell infiltrations with PUUV antigen presence in capillary vascular ECs and mononuclear cells in the lung (Rasmuson et al., 2011; Clement et al., 2014).

### Heart

Cardiovascular disorders are identified as the leading cause of death during or shortly after PUUV infection (Connolly-Andersen et al., 2013). Although PUUV infection has a relatively low case fatality rate, cardiopulmonary complications can have implications on the recovery of a majority of patients (Rasmuson et al., 2013). Implications may consist of increased left ventricular stroke volume and myocardial contraction causing delayed functional hemodynamical recovery. During active NE, sinus bradycardia, T-wave inversion, and ST segment changes are described as common electrocardiographic (ECG) findings (Puljiz et al., 2005; Kitterer et al., 2016). However, these ECG abnormalities were transient in almost all of the patients and were not associated with negative cardiovascular outcome. Unfortunately, none of these studies specified viral antigen presence in cardiac cells. However, another case report specifically mentioned that heart tissue samples were negative for PUUV antigen (Hautala et al., 2002). Since there is no evidence of infection in the heart tissue, increased myocardial energy demand seems to be result of permeability increases of peripheral blood vessels.

### Kidneys

While the exact mechanism of extrapulmonary spread remains unknown, once the virus reaches the vasculature there is a potential for rapid systemic dissemination. Following entry via the respiratory tract, the kidneys are considered the primary target for HFRS viruses. Renal function is dependent on the integrity of tubular epithelium and the glomeruli, which predominantly consist of fenestrated ECs, podocytes, and basement membrane. The disease severity of HFRS (including NE) ranges from reversible mild to severe acute kidney injury (Jonsson et al., 2010; Mustonen et al., 2017). In severe cases, oliguria, severe interstitial edema and hemorrhages are common clinical manifestations and hemodialysis may be required (Kim et al., 1993; Suh et al., 1995; Hautala et al., 2002; Jonsson et al., 2010). Patients can typically display acute interstitial inflammation, tubulointerstitial nephritis with focal interstitial hemorrhages (Collan et al., 1991; Kim et al., 1993; Groen et al., 1996; Temonen et al., 1996; Sironen et al., 2008; Meier et al., 2018). In addition to tubular and glomerular capillary ECs, HTNV and PUUV antigens have been detected in the tubular epithelial cells of HFRS patients (Hung et al., 1992; Kim et al., 1993; Groen et al., 1996; Krautkramer et al., 2011, 2013). Acute necrosis of antigen-positive tubular epithelium and the presence of tubular epithelial cells in urine (Kim et al., 1993; Hautala et al., 2002) suggest that–in addition to EC dysfunction–tubular damage contributes to kidney function impairment in HFRS (Hung et al., 1992). *In vitro* studies have demonstrated that orthohantavirus-induced interstitial nephritis can be distinguished from non-orthohantavirus-induced interstitial nephritis due to signs of redistribution of tight junction proteins (e.g., ZO-1) in glomerular and tubular cells (Krautkramer et al., 2011). Decreased glomerular ZO-1 expression may also result in reduced function of the glomerulus as a molecular filter by enhancing glomerular permeability (Krautkramer et al., 2011). Finally, nucleocapsid (N) proteins of HTNV and PUUV cause impairment of podocyte motility and adhesion capacity (Hagele et al., 2018). Infection of podocytes leads to virus-induced cytoskeletal rearrangements *in vitro*, which could indicate a role for podocyte foot process effacement in observed proteinuria *in vivo* (Boehlke et al., 2014; Hagele et al., 2019). These rearrangements are more prominent for HTNV compared to PUUV, which corresponds with more pronounced proteinuria and kidney injury as observed during HFRS (Hagele et al., 2019).

### Liver

Involvement of the liver has mostly been reported in SEOV cases (Kim et al., 1995; Zhang et al., 2011), where it results in acute viral hepatitis-like manifestations with lobular necrosis without viral inclusions, atypical cells, vasculitis, or fibrosis, a painful enlarged liver and distinct elevation of liver enzymes (Kim et al., 1995; Nielsen et al., 2010; Swanink et al., 2018). In addition, focal midzonal necrosis associated with mild mononuclear infiltrates can be observed in the liver during HTNV infection (Elisaf et al., 1993). However, detection of viral antigen in liver tissues has not been specifically reported in HFRS cases. Therefore, elevated liver enzymes seem to be a consequence of inflammatory events rather than direct infection.

### Spleen

The spleen contains approximately one third of the body's platelets content illustrating its role in controlling the balance of available blood platelets and hence preventing thrombocytopenia (Bassenge, 1996). Data from a limited number of severe NE patients demonstrates venous congestion, splenomegaly, and variable amounts of antigen-positive ECs in the spleen, presumably sinusoidal lining cells (Hautala et al., 2002; Koskela et al., 2014; Sironen et al., 2017). However, to date, there is no association between enhanced sequestration of blood platelets in the spleen and the pathogenesis of thrombocytopenia during HFRS/NE (Koskela et al., 2014). The most likely explanation of the pathogenesis of thrombocytopenia during HFRS/NE, seems to be peripheral consumption (adherence to ECs), since several bone marrow studies showed a normal morphogenesis of platelets (Lee, 1987; Lutteke et al., 2010).

## HCPS in Humans and Disease Models

### Conducting Airways

**Figure 2:**
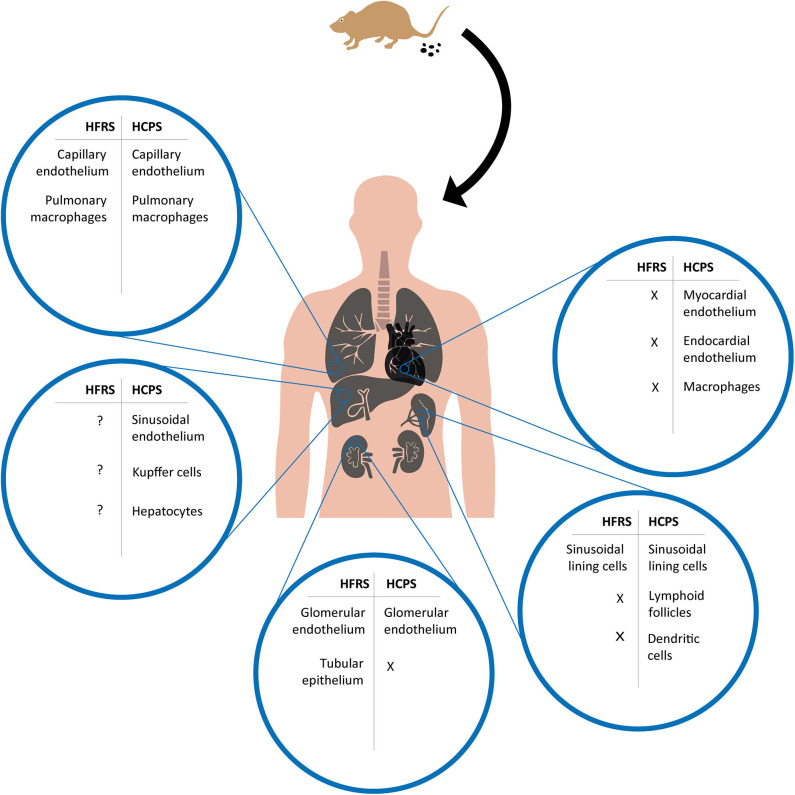
Overview of cell tropism during HFRS and HCPS based on human and experimental disease models. After a human host is infected by inhalation of virus containing aerosolized excreta of an infected rodent, orthohantavirus is able to reach multiple organs and infect different cell types. Potentially infected cell types during HFRS and HCPS are compared for major organs in which viral antigens have been detected in human tissues or experimental disease models; lungs, heart, kidneys, liver, and spleen. X = absence of viral antigen; ? = viral antigen presence not specified.

In comparison to HFRS, orthohantavirus spread has been described in a wider range of organs for HCPS (Figure 2). Similar to HFRS, SNV RNA was found at low abundance in tracheal aspirate in a small number of HCPS patients (Xiao et al., 2006). Unlike other orthohantaviruses, ANDV is associated with human-to-human transmission. Efficient infection of the upper respiratory tract favors host-to-host transmission, as has been shown for other respiratory viruses, like influenza (van Riel et al., 2010), suggesting a potential role for respiratory cell infection. This is in line with data from an experimental ANDV infection model in Syrian hamsters in which viral antigen was detected in tracheal tissues following intranasal challenge (Safronetz et al., 2011). This antigen staining was only focal with limited spread to neighboring cells, and no observed histological abnormalities (Safronetz et al., 2011). *In vitro*, ANDV infects non-ciliated cells (e.g., club and goblet cells) resulting in bidirectional virus release, which could facilitate direct access to infect adjacent respiratory epithelium or systemic spread by infection of respiratory ECs (Rowe and Pekosz, 2006). The exact role of infected non-ciliated cells during initial stages of disease in humans remains to be determined.

### Lungs

Since (exudative) thoracic effusions and pulmonary edema are classical hallmarks of HCPS (Duchin et al., 1994; Hallin et al., 1996), pulmonary involvement has been studied to a great extent. Severe SNV infection in HCPS patients causes interstitial pneumonitis with variable mononuclear cell infiltrates, pulmonary edema, and focal hyaline membranes (Zaki et al., 1995). SNV infection leads to an increase of plasminogen activator inhibitor type 1 (PAI-1) in plasma samples of terminal stage patients (Bondu et al., 2018). Upregulation of this fibrinolysis inhibitor may lead to excessive fibrin accumulation, explaining the observed focal hyaline membranes in lungs of HCPS patients (Zaki et al., 1995). In contrast to other respiratory viral infections–like influenza (Kuiken and Taubenberger, 2008)–there is no cellular debris of respiratory epithelial cells and/or type II pneumocyte hyperplasia. This suggests that viral spread to the circulation does not rely on disrupted epithelial layers. Based on samples from end stages of disease, antigens from HCPS causing viruses are predominantly detected in the ECs of small vessels in the lungs and macrophages with almost no cells that remain unaffected (Nolte et al., 1995; Zaki et al., 1995; Green et al., 1998; Toro et al., 1998). Viral antigen was typically not detected in the ECs of large blood vessels in humans (Zaki et al., 1995). Furthermore, high viral load in lung tissue is usually negatively correlated to survival of patients (Zaki et al., 1995). Cell types that may play a role during the early stages of pathogenesis remain unknown due to the lack of early samples from the lungs.

### Heart

Cardiac involvement varies in HCPS patients, ranging from mild forward failure with stable blood flow to fulminant shock and rapid death (Bustamante et al., 1997). Severe cases initially display signs of increased vascular permeability leading to non-cardiogenic pulmonary edema which later develops into cardiac complications (Hallin et al., 1996; Peters et al., 1999). In contrast to HFRS viruses, viral antigen is detected in ECs and macrophages in the myocardium and sporadically the endocardium of SNV-infected patients (Nolte et al., 1995; Zaki et al., 1995; Green et al., 1998; Saggioro et al., 2007). It is believed that direct infection of cardiac tissue (combined with existing pulmonary edema) can lead to cardiac remodeling (flabby wall and mild biventricular dilatation), scattered foci of myofiber necrosis and a mild to moderate interstitial edema with mono-nuclear infiltrate (Saggioro et al., 2007). This likely causes an atypical form of cardiogenic shock by myocardial dysfunction associated to myocarditis (Saggioro et al., 2007), which can lead to decreased tissue perfusion, metabolic acidosis, and malignant arrhythmias (Hjelle, 2002). Clinical studies have identified impaired myocardial function instead of hypoxic injury as a leading cause of death in HCPS (Duchin et al., 1994; Hallin et al., 1996).

### Liver

Clinical and post-mortem data from HCPS patients suggest that liver contribution to pathogenesis is limited. Still, viral antigen (predominantly SNV) can sporadically be detected in hepatocytes, sinusoidal ECs, and Kupffer cells (Zaki et al., 1995; Green et al., 1998; Toro et al., 1998). In addition, infiltration of mononuclear inflammatory cells is observed (Nolte et al., 1995; Zaki et al., 1995), like in other organs (e.g., lungs and heart) without histopathology (Zaki et al., 1995).

### Kidneys

The lungs are the primary target organ during HCPS, and the majority of HCPS reports actually do not study renal effects. Although a case report showed predominantly renal staining for SNV antigen, suggesting that renal tropism may overrule general pulmonary involvement in SNV infection (Passaro et al., 2001). Additionally, renal symptoms such as polyuria and proteinuria are common findings among a minority of HCPS patients (Jonsson et al., 2010; Clement et al., 2014). A frequent cause for these symptoms is increased glomerular capillary permeability to protein. Accordingly, widespread presence of SNV antigen in glomerular capillary ECs can be detected (Zaki et al., 1995; Green et al., 1998). It is plausible that infection of glomerular ECs during HCPS does not lead to clinical signs as more prominent pulmonary symptoms might arise earlier. Alternatively, tubular reabsorption could also compensate for decreased glomerular filter function, as tubular epithelium is negative for infection by HCPS-causing viruses (Zaki et al., 1995; Green et al., 1998).

### Spleen

Mild splenomegaly with atypical mononuclear cells in red pulp and periarteriolar sheaths of the white pulp are common findings in later stages of HCPS (Nolte et al., 1995; Zaki et al., 1995). The white and red pulp of the spleen house a great variety of cell types, such as monocytes, lymphocytes, and dendritic cells. Viral antigen varies from negative to widespread and can be detected in multiple cell types, such as vascular ECs, lymphoid follicles, and splenic dendritic cells (Zaki et al., 1995; Green et al., 1998; Hooper et al., 2001). These data suggest that in addition to splenic ECs, immune cells can be infected during HCPS (e.g., SNV infection). As an essential location of mononuclear phagocyte system activity, viral replication in immune cells might provide an essential route for viral dissemination throughout the body. Altogether, these data suggest that the role of the spleen during HCPS might be more prominent than previously considered.

## HFRS/NE-Associated Viruses in Reservoir Rodents

### Respiratory Tract

Similar to transmission to humans, animal-to-animal transmission in the reservoir host is assumed to occur primarily via the respiratory route. However, unlike in humans, infection in reservoir species results in persistent infection without clinical signs. To our knowledge, no studies have focused on the presence of virus in the conducting airways of orthohantavirus reservoir rodents. So, it remains unclear which cells from the upper respiratory tract can be infected by HFRS viruses in reservoir rodents. Nevertheless, lung tissues are frequently screened for surveillance of orthohantaviruses in reservoir species as highest antigen concentrations can be found here (Lee et al., 1982). Since the Norway rat (*Rattus norvegicus*) is a model organism for different diseases with many reagents available, SEOV infection in reservoir rodents has been studied more extensively compared to other HFRS viruses (Easterbrook and Klein, 2008a). The lungs are considered the primary site of viral replication during persistent and experimental infection of SEOV in Norway rats (Easterbrook and Klein, 2008b). Following intraperitoneal inoculation of SEOV, viral antigen was mainly detected in pulmonary ECs and alveolar macrophages during persistence (i.e., defined as ≥30 days post-infection) (Easterbrook and Klein, 2008b). Although this entry route is distinct from what is expected during natural infection, pulmonary cells are also considered a target early in natural infection. In naturally infected rats, SEOV antigen is indeed primarily detected in interstitial ECs of alveolar septal capillaries, and rarely in ECs of larger blood vessels such as pulmonary veins, similar to human infection (Maas et al., 2019).

### Heart

In addition to the lungs, in one study, DOBV and TULV could be detected by polymerase chain reaction (PCR) in hearts of naturally infected animal reservoirs (Michalski et al., 2014). Unfortunately, this study did not identify the infected cell types. Analogous to human data, PUUV is generally absent in cardiac cells of naturally infected reservoir voles (Michalski et al., 2014; Dervovic and Hukic, 2016). Consequently, virus presence in cardiac cells could depend on the specific causative virus.

### Kidneys

As the kidneys are the main target organ during HFRS in humans, it is important to identify which (immunological) mechanisms prevent this renal pathology in the reservoir and thus which renal cell types are infected in healthy reservoir animals (Easterbrook and Klein, 2008a). Almost 40 years ago, the first HFRS orthohantaviral antigens were reported in kidneys of wild rodents (Lee et al., 1982; LeDuc et al., 1984). To our knowledge, the infected renal cell types have not been specified in reservoir rodents, other than SEOV in microvascular ECs (Maas et al., 2019). However, HFRS/NE-associated orthohantaviruses are commonly detected in urine of reservoir hosts (Lee et al., 1981; Gavrilovskaya et al., 1983; Yanagihara et al., 1985; Klein et al., 2001; Hardestam et al., 2008; Voutilainen et al., 2015). Orthohantaviruses are much larger than the size of filterable macromolecules. This indicates that viruria is a consequence of viral particles release from the apical membranes of infected renal cells or disruption of glomerular filter function.

### Liver

PUUV antigen can be detected in the liver of a minority (4%) of naturally infected wild rodent reservoir hosts (Gavrilovskaya et al., 1983). Intramuscular infections of PUUV in bank voles results in viral antigen in liver ECs and Kupffer cells (Yanagihara et al., 1985). Interestingly, natural SEOV infection in rats primarily targets the microvasculature in the liver and results in a mild hepatitis, characterized by an increase in the number of polymorphonuclear cells within the hepatic parenchyma and sinusoids (Maas et al., 2019). This suggests that SEOV infects hepatic ECs in both reservoir and diseased host.

### Spleen

The spleen is a peripheral immune organ that supports merely low levels of virus replication in reservoir rodents (Lee et al., 1982; Gavrilovskaya et al., 1983; LeDuc et al., 1984; Yanagihara et al., 1985; Compton et al., 2004; Michalski et al., 2014). Although evidence is conflicting and cell types are not consistently specified, splenic endothelium and macrophages seem to be the main target cells (Yanagihara et al., 1985; Maas et al., 2019). During both acute and persistent SEOV infection in spleen tissues, proinflammatory (e.g., IL-1β, IL-6, and TNF-α) and antiviral responses (e.g., IFN-γ) are elevated to stimulate viral clearance, while regulatory responses (e.g., TGF-β) seem unaltered (Easterbrook and Klein, 2008b). These data differ from local immune reactions in the lungs, where regulatory responses are elevated (Easterbrook et al., 2007; Easterbrook and Klein, 2008b). Altogether, these data suggest that local shifts in the immunological balance might be crucial for controlling virus replication, hence preventing pathogenesis.

## HCPS-Associated Viruses in Reservoir Rodents

### Respiratory Tract

In naturally infected deer mice, the reservoir rodents of SNV, the highest levels of virus can be detected in the lungs (Netski et al., 1999). Mild lung pathology is observed in the majority of wild rodents infected by SNV, as characterized by alveolar septal edema with various levels of SNV antigen in the alveolar and capillary walls (Netski et al., 1999). A transmission study in reservoir rodents naturally infected with ANDV demonstrated viral antigen in most of the epithelium lining the alveoli and some of the capillary ECs (Padula et al., 2004). These observations differ from end stage human infections, during which microvascular ECs are primarily infected (Zaki et al., 1995; Green et al., 1998; Toro et al., 1998). Differences in viral spread could depend on the host's specific ability to clear the virus, differential distribution of the viral entry receptors and the stage of infection, since most data on cell tropism from human cases is based on the end stage of disease (Billings et al., 2010).

### Heart

In cardiac tissues of SNV-infected deer mice, only few antigen positive cells can be detected (Green et al., 1998; Botten et al., 2002). Therefore, prominent infection of cardiac tissue with consequent disease manifestations seems specific for human infections, at least for SNV infection (Nolte et al., 1995; Zaki et al., 1995; Green et al., 1998; Saggioro et al., 2007).

### Liver

SNV infection results in immune infiltrates in the hepatic portal zones of reservoir hosts (Netski et al., 1999). These immune infiltrations in infected liver tissue are the second most observed pathological finding after alveolar septal edema in wild deer mice (Netski et al., 1999). These data again imply that SNV is able to cause pathology within reservoir hosts. However, it remains to be confirmed whether infiltration of immune cells leads to liver function impairment in wild deer mice. As SNV antigen can be found in infiltrating mononuclear cells, Kupffer cells in liver sinuses and hepatocytes (Netski et al., 1999), liver infection by other HCPS orthohantaviruses in their reservoir species should be monitored to exclude the possibility that these observations are specific for persistent SNV infection.

### Kidneys

In deer mice, naturally infected with SNV, no gross kidney pathology is observed. Furthermore, focal to no viral antigen can be detected in kidneys (specifically in glomerular tissue) (Green et al., 1998; Netski et al., 1999). It has been described that the highest levels of virus in urine are shed during earlier stages of infection (Netski et al., 1999).

### Spleen

SNV antigen can be detected in mononuclear cells within both red and white pulp of the spleen of wild deer mice in one study (Netski et al., 1999), but not in another (Green et al., 1998). Contradictions between these studies may likely be explained by the unknown timing of natural infection. It remains to be determined whether infection of (immune) cells in the spleen plays an important role in viral dissemination and which local mechanisms aid to persistent infection as opposed to pathogenesis in humans.

## Conclusion and Future Perspective

While human orthohantaviruses enter the host via the respiratory tract, it remains unknown which cells in the human respiratory tract are the first infected. It has been described that orthohantaviruses are endotheliotropic viruses, however this review demonstrates that additional cell types are infected, which may play a role in the pathogenesis of these viruses (Table 1).

**Table 1:** Organ-specific cell types contributing to orthohantavirus disease *in vivo* summarized for five major organs.

**Affected organ**	**Infected cell type**	**HFRS**		**HCPS**		**Hypothesis on pathology**	**References**
		**Human**	**Reservoir**	**Human**	**Reservoir**		
Lungs	Pulmonary microvascular endothelium	+	+	+	+	Extensive infection leads to immune cell infiltrations and endothelial cell activation, which causes local inflammation and pulmonary edema	Brummer-Korvenkontio et al., 1980; Lee et al., 1981, 1982; Gavrilovskaya et al., 1983; LeDuc et al., 1984; Yanagihara et al., 1985; Nolte et al., 1995; Zaki et al., 1995; Green et al., 1998; Toro et al., 1998; Netski et al., 1999; Padula et al., 2004; Easterbrook and Klein, 2008b; Rasmuson et al., 2011; Clement et al., 2014
Heart	Myocardial endothelium	–	?	+	+	Infection leads to immune cell infiltrations and endothelial cell activation, causing interstitial edema that contributes to myocardial dysfunction and cardiogenic shock	Nolte et al., 1995; Zaki et al., 1995; Green et al., 1998; Botten et al., 2002; Hautala et al., 2002; Saggioro et al., 2007; Michalski et al., 2014; Dervovic and Hukic, 2016
Kidneys	Tubular epithelium	+	+^*^	–	?	Infection of endothelium leads to immune cell infiltrations (tubulointerstitial nephritis) with redistribution of tight junction proteins, along with direct tubular necrosis (with possible interstitial hemorrhages) causing functional impairment of tubuli leading to proteinuria, microscopic hematuria	Hung et al., 1992; Kim et al., 1993; Groen et al., 1996; Green et al., 1998; Botten et al., 2002; Hautala et al., 2002; Krautkramer et al., 2011
	Glomerular endothelium	+	+^*^	+	+	Infection of glomeruli causes decrease in glomerular ZO-1 expression relating to reduced function of the glomerulus as molecular filter by enhancing glomerular permeability, leading to proteinuria and microscopic hematuria	Zaki et al., 1995; Groen et al., 1996; Green et al., 1998; Netski et al., 1999; Botten et al., 2002; Krautkramer et al., 2013
Liver	Hepatic sinusoidal endothelium	?	+	+	+	Infection of endothelium leads to immune cell infiltrations (antigen-positive Kupffer cells) and increased vascular permeability, which probably do not lead to significant liver dysfunction as hepatic sinusoidal microvasculature is already relatively permeable	Gavrilovskaya et al., 1983; Yanagihara et al., 1985; Zaki et al., 1995; Green et al., 1998; Toro et al., 1998; Netski et al., 1999
Spleen	Splenic sinusoidal endothelium	+	+	+	+^*^	Infection of immune cells in the spleen may cause over-activation of immature lymphocytes elsewhere and facilitate prolonged virus dissemination throughout the body	Lee et al., 1982; Gavrilovskaya et al., 1983; LeDuc et al., 1984; Yanagihara et al., 1985; Zaki et al., 1995; Green et al., 1998; Netski et al., 1999; Hautala et al., 2002; Klingstrom et al., 2002; Compton et al., 2004; Padula et al., 2004; Sironen et al., 2008, 2017; Michalski et al., 2014

Viral antigen presence in mononuclear immune cells are not included in table. + = viral antigen present of at least one causative virus species, ? = conflicting data/not tested, – = viral antigen absent of all tested causative virus species, and ^*^ = studies did not specify infected cell type within organ.

Although the initial target cells are unknown, ECs are an important target later during infection. Interestingly, orthohantavirus infection of ECs does not result in overt cell damage, and infected ECs can be found in most organs. However, orthohantavirus induced pathology is only observed in specific organs, believed to play a key role in pathogenesis, including, lung (HCPS), and kidneys (HFRS). Unlike in humans, orthohantavirus infection in the reservoir host causes a persistent infection with limited pathological changes and no apparent clinical signs. Unraveling the pathogenesis of orthohantaviruses (or any emerging virus) through patient-based research is extremely difficult. Additionally, our understanding of the differential pathogenesis of orthohantaviruses in humans has been hampered by the lack of relevant animal models that allow the comparison of HFRS- and HCPS-causing viruses, the limited availability of *in vivo* and *in vitro* models of the reservoir host, and the requirement of high containment facilities for orthohantaviruses pathogenic to humans.

As reviewed above, ECs of the microvasculature in multiple organs are the main targets for orthohantaviruses both in the reservoir hosts and humans. Here we provide an overview of additional cells targeted by orthohantaviruses in the respiratory tract, heart, kidneys, liver, and spleen and the potential role they play in pathogenesis. These organ systems were chosen as multiple studies have demonstrated viral presence in these organs in human cases. While there are a variety of studies discussing orthohantavirus infection in other organs, such as intestines (Zaki et al., 1995; Green et al., 1998; Latus et al., 2014), endocrine system (Zaki et al., 1995; Green et al., 1998; Bhoelan et al., 2019), and brain (Zaki et al., 1995) in humans, but also brown adipose tissue (Botten et al., 2002) in reservoir rodents, these were not included due to lack of mechanistic studies. Of note, transmission via saliva is suggested to be even more relevant than urine among naturally infected hosts as SEOV and ANDV have been detected more often in either saliva (and salivary glands) compared to urine samples (Padula et al., 2004; Maas et al., 2019).

So far, it remains unknown how viral dissemination occurs in an infected host post-inhalation. Potential mechanisms include initial infection of respiratory epithelium and either basolateral release or cell-to-cell spread to ECs to reach the circulation, as shown for other respiratory viruses, like measles (Singh et al., 2016). Alternatively, infection of immune cells in the respiratory tract could facilitate systemic spread via the vascular and lymphatic system, as described for another hemorrhagic fever virus; Ebola virus (Bray and Geisbert, 2005).

Interestingly, distinct orthohantavirus species seem to cause different degrees of pathology in various organs. While the use of α_v_β_3_ integrins and other (co-)receptors do correlate with pathogenicity in humans, distribution of these viral receptors on human cells does not correspond with the susceptibility and organ tropism of orthohantavirus infection *in vivo*. Therefore, the exploration of additional host cell (co-)receptors that facilitate orthohantavirus entry and/or attachment *in vivo* should continue. In addition, it remains interesting that during HCPS and HFRS ECs of almost all major organs are affected, and yet the clinical signs per organ generally seem to differ per causative virus species, although exceptions have been reported. Therefore, effects of infection by various orthohantaviruses on organ-specific microvascular ECs should be explored. Moreover, the pathogenic mechanisms occurring in other cell types that are infected during HFRS and not HCPS (and vice versa) could also be at the base of understanding why HFRS and HCPS mainly lead to, respectively kidney and lung complications, for instance the potential role of tubular epithelium in kidney disease. In a broader sense, the conclusion that orthohantaviruses cause disease in humans and generally not in their reservoir hosts, while targeting similar cells and organs provides a unique opportunity to identify key host factors that play a role the in the observed host-specific pathogenesis. Altogether, addressing these research questions will aid in our understanding of orthohantavirus pathogenesis and will be instrumental in identifying potential therapeutic and prophylactic strategies.

## Author Contributions

DN and BR contributed to the organization and structure of the review. DN performed the literature survey and prepared the draft. DN, MG, CR, MK, and BR contributed to critical evaluation and finalizing of the manuscript. All authors contributed to the article and approved the submitted version.

## Conflict of Interest

The authors declare that the research was conducted in the absence of any commercial or financial relationships that could be construed as a potential conflict of interest.

## References

[B1] Abudurexiti A. Adkins S. Alioto D. Alkhovsky S. V. Avsic-Zupanc T. Ballinger M. J.. (2019). Taxonomy of the order Bunyavirales: update 2019. Arch. Virol. 164, 1949–1965. 10.1007/s00705-019-04253-6 31065850 PMC6641860

[B2] Alonso D. O. Iglesias A. Coelho R. Periolo N. Bruno A. Cordoba M. T.. (2019). Epidemiological description, case-fatality rate, and trends of Hantavirus Pulmonary Syndrome: 9 years of surveillance in Argentina. J. Med. Virol. 91, 1173–1181. 10.1002/jmv.25446 30840775

[B3] An S. S. Pennella C. M. Gonnabathula A. Chen J. Wang N. Gaestel M.. (2005). Hypoxia alters biophysical properties of endothelial cells via p38 MAPK- and Rho kinase-dependent pathways. Am. J. Physiol. Cell Physiol. 289, 521–530. 10.1152/ajpcell.00429.2004 15857906

[B4] Antonen J. Leppanen I. Tenhunen J. Arvola P. Makela S. Vaheri A.. (2013). A severe case of Puumala hantavirus infection successfully treated with bradykinin receptor antagonist icatibant. Scand. J. Infect. Dis. 45, 494–496. 10.3109/00365548.2012.755268 23294035

[B5] Avraamides C. J. Garmy-Susini B. Varner J. A. (2008). Integrins in angiogenesis and lymphangiogenesis. Nat. Rev. Cancer 8, 604–617. 10.1038/nrc2353 18497750 PMC2577722

[B6] Bassenge E. (1996). Endothelial function in different organs. Prog. Cardiovasc. Dis. 39, 209–228. 10.1016/S0033-0620(96)80002-8 8970574

[B7] Bates D. O. (2010). Vascular endothelial growth factors and vascular permeability. Cardiovasc. Res. 87, 262–271. 10.1093/cvr/cvq105 20400620 PMC2895541

[B8] Bhoelan S. Langerak T. Noack D. van Schinkel L. van Nood E. van Gorp E. C. M.. (2019). Hypopituitarism after orthohantavirus infection: what is currently known? Viruses 11:340. 10.3390/v11040340 30974852 PMC6521286

[B9] Billings A. N. Rollin P. E. Milazzo M. L. Molina C. P. Eyzaguirre E. J. Livingstone W.. (2010). Pathology of Black Creek Canal virus infection in juvenile hispid cotton rats (*Sigmodon hispidus*). Vector Borne Zoonotic Dis. 10, 621–628. 10.1089/vbz.2009.0156 20455779 PMC2979341

[B10] Bird B. H. Shrivastava-Ranjan P. Dodd K. A. Erickson B. R. Spiropoulou C. F. (2016). Effect of Vandetanib on Andes virus survival in the hamster model of Hantavirus pulmonary syndrome. Antiviral Res. 132, 66–69. 10.1016/j.antiviral.2016.05.014 27233645

[B11] Boehlke C. Hartleben B. Huber T. B. Hopfer H. Walz G. Neumann-Haefelin E. (2014). Hantavirus infection with severe proteinuria and podocyte foot-process effacement. Am. J. Kidney Dis. 64, 452–456. 10.1053/j.ajkd.2014.04.030 24954247

[B12] Bondu V. Bitting C. Poland V. L. Hanson J. A. Harkins M. S. Lathrop S.. (2018). Upregulation of P2Y2R, active uPA, and PAI-1 are essential components of hantavirus cardiopulmonary syndrome. Front. Cell. Infect. Microbiol. 8:169. 10.3389/fcimb.2018.00169 29930915 PMC6001748

[B13] Bondu V. Wu C. Cao W. Simons P. C. Gillette J. Zhu J.. (2017). Low-affinity binding in cis to P2Y2R mediates force-dependent integrin activation during hantavirus infection. Mol. Biol. Cell 28, 2887–2903. 10.1091/mbc.e17-01-0082 28835374 PMC5638590

[B14] Botten J. Mirowsky K. Ye C. Gottlieb K. Saavedra M. Ponce L.. (2002). Shedding and intracage transmission of Sin Nombre hantavirus in the deer mouse (*Peromyscus maniculatus*) model. J. Virol. 76, 7587–7594. 10.1128/JVI.76.15.7587-7594.2002 12097572 PMC136373

[B15] Bray M. Geisbert T. W. (2005). Ebola virus: the role of macrophages and dendritic cells in the pathogenesis of Ebola hemorrhagic fever. Int. J. Biochem. Cell Biol. 37, 1560–1566. 10.1016/j.biocel.2005.02.018 15896665

[B16] Brummer-Korvenkontio M. Vaheri A. Hovi T. von Bonsdorff C. H. Vuorimies J. Manni T.. (1980). Nephropathia epidemica: detection of antigen in bank voles and serologic diagnosis of human infection. J. Infect. Dis. 141, 131–134. 10.1093/infdis/141.2.131 6102587

[B17] Bustamante E. A. Levy H. Simpson S. Q. (1997). Pleural fluid characteristics in hantavirus pulmonary syndrome. Chest 112, 1133–1136. 10.1378/chest.112.4.1133 9377934

[B18] Chi J. T. Chang H. Y. Haraldsen G. Jahnsen F. L. Troyanskaya O. G. Chang D. S.. (2003). Endothelial cell diversity revealed by global expression profiling. Proc. Natl. Acad. Sci. U.S.A. 100, 10623–10628. 10.1073/pnas.1434429100 12963823 PMC196854

[B19] Choi Y. Kwon Y. C. Kim S. I. Park J. M. Lee K. H. Ahn B. Y. (2008). A hantavirus causing hemorrhagic fever with renal syndrome requires gC1qR/p32 for efficient cell binding and infection. Virology 381, 178–183. 10.1016/j.virol.2008.08.035 18834607

[B20] Clement J. Colson P. Mc Kenna P. (1994). Hantavirus pulmonary syndrome in New England and Europe. N. Engl. J. Med. 331, 545–546; author reply 547–548. 10.1056/NEJM199408253310813 8041425

[B21] Clement J. Maes P. Van Ranst M. (2014). Hemorrhagic fever with renal syndrome in the new, and hantavirus pulmonary syndrome in the old world: paradi(se)gm lost or regained? Virus Res. 187, 55–58. 10.1016/j.virusres.2013.12.036 24440318

[B22] Collan Y. Mihatsch M. J. Lahdevirta J. Jokinen E. J. Romppanen T. Jantunen E. (1991). Nephropathia epidemica: mild variant of hemorrhagic fever with renal syndrome. Kidney Int. Suppl. 35, 62–71. 1685202

[B23] Compton S. R. Jacoby R. O. Paturzo F. X. Smith A. L. (2004). Persistent Seoul virus infection in Lewis rats. Arch. Virol. 149, 1325–1339. 10.1007/s00705-004-0294-y 15221534 PMC7087218

[B24] Connolly-Andersen A. M. Ahlm K. Ahlm C. Klingstrom J. (2013). Puumala virus infections associated with cardiovascular causes of death. Emerg. Infect. Dis. 19, 126–128. 10.3201/eid1901.111587 23260342 PMC3557968

[B25] Connolly-Andersen A. M. Sundberg E. Ahlm C. Hultdin J. Baudin M. Larsson J.. (2015). Increased thrombopoiesis and platelet activation in hantavirus-infected patients. J. Infect. Dis. 212, 1061–1069. 10.1093/infdis/jiv161 25762786

[B26] Dalrymple N. A. Mackow E. R. (2014). Virus interactions with endothelial cell receptors: implications for viral pathogenesis. Curr. Opin. Virol. 7, 134–140. 10.1016/j.coviro.2014.06.006 25063986 PMC4206553

[B27] de Oliveira R. C. Guterres A. Fernandes J. D'Andrea P. S. Bonvicino C. R. de Lemos E. R. (2014). Hantavirus reservoirs: current status with an emphasis on data from Brazil. Viruses 6, 1929–1973. 10.3390/v6051929 24784571 PMC4036540

[B28] Dehler M. Zessin E. Bartsch P. Mairbaurl H. (2006). Hypoxia causes permeability oedema in the constant-pressure perfused rat lung. Eur. Respir. J. 27, 600–606. 10.1183/09031936.06.00061505 16507862

[B29] Dervovic E. Hukic M. (2016). Detection of Puumala virus in the tissue of infected naturally rodent hosts in the area of central Dinarides. J. Virol. Methods 230, 24–27. 10.1016/j.jviromet.2016.01.007 26800777

[B30] Duchin J. S. Koster F. T. Peters C. J. Simpson G. L. Tempest B. Zaki S. R.. (1994). Hantavirus pulmonary syndrome: a clinical description of 17 patients with a newly recognized disease. The Hantavirus Study Group. N. Engl. J. Med. 330, 949–955. 10.1056/NEJM199404073301401 8121458

[B31] Dvorak H. F. (2010). Vascular permeability to plasma, plasma proteins, and cells: an update. Curr. Opin. Hematol. 17, 225–229. 10.1097/MOH.0b013e3283386638 20375889 PMC2878124

[B32] Easterbrook J. D. Klein S. L. (2008a). Immunological mechanisms mediating hantavirus persistence in rodent reservoirs. PLoS Pathog. 4:e1000172. 10.1371/journal.ppat.1000172 19043585 PMC2584234

[B33] Easterbrook J. D. Klein S. L. (2008b). Seoul virus enhances regulatory and reduces proinflammatory responses in male Norway rats. J. Med. Virol. 80, 1308–1318. 10.1002/jmv.21213 18461618 PMC4145243

[B34] Easterbrook J. D. Zink M. C. Klein S. L. (2007). Regulatory T cells enhance persistence of the zoonotic pathogen Seoul virus in its reservoir host. Proc. Natl. Acad. Sci. U.S.A. 104, 15502–15507. 10.1073/pnas.0707453104 17878294 PMC2000529

[B35] Elisaf M. Stefanaki S. Repanti M. Korakis H. Tsianos E. Siamopoulos K. C. (1993). Liver involvement in hemorrhagic fever with renal syndrome. J. Clin. Gastroenterol. 17, 33–37. 10.1097/00004836-199307000-00010 8104972

[B36] Gavard J. (2014). Endothelial permeability and VE-cadherin: a wacky comradeship. Cell Adh. Migr. 8, 158–164. 10.4161/cam.29026 25422846 PMC4049861

[B37] Gavrilovskaya I. Gorbunova E. Koster F. Mackow E. (2012). Elevated VEGF levels in pulmonary edema fluid and PBMCs from patients with acute hantavirus pulmonary syndrome. Adv. Virol. 2012:674360. 10.1155/2012/674360 22956954 PMC3432326

[B38] Gavrilovskaya I. N. Apekina N. S. Myasnikov Yu A. Bernshtein A. D. Ryltseva E. V. Gorbachkova E. A.. (1983). Features of circulation of hemorrhagic fever with renal syndrome (HFRS) virus among small mammals in the European U.S.S.R. Arch. Virol. 75, 313–316. 10.1007/BF01314898 6220688

[B39] Gavrilovskaya I. N. Brown E. J. Ginsberg M. H. Mackow E. R. (1999). Cellular entry of hantaviruses which cause hemorrhagic fever with renal syndrome is mediated by beta3 integrins. J. Virol. 73, 3951–3959. 10.1128/JVI.73.5.3951-3959.1999 10196290 PMC104173

[B40] Gavrilovskaya I. N. Gorbunova E. E. Mackow E. R. (2010). Pathogenic hantaviruses direct the adherence of quiescent platelets to infected endothelial cells. J. Virol. 84, 4832–4839. 10.1128/JVI.02405-09 20181715 PMC2863738

[B41] Gavrilovskaya I. N. Gorbunova E. E. Mackow E. R. (2013). Hypoxia induces permeability and giant cell responses of Andes virus-infected pulmonary endothelial cells by activating the mTOR-S6K signaling pathway. J. Virol. 87, 12999–13008. 10.1128/JVI.02103-13 24067973 PMC3838155

[B42] Gavrilovskaya I. N. Gorbunova E. E. Mackow N. A. Mackow E. R. (2008). Hantaviruses direct endothelial cell permeability by sensitizing cells to the vascular permeability factor VEGF, while angiopoietin 1 and sphingosine 1-phosphate inhibit hantavirus-directed permeability. J. Virol. 82, 5797–5806. 10.1128/JVI.02397-07 18367532 PMC2395149

[B43] Gavrilovskaya I. N. Peresleni T. Geimonen E. Mackow E. R. (2002). Pathogenic hantaviruses selectively inhibit beta3 integrin directed endothelial cell migration. Arch. Virol. 147, 1913–1931. 10.1007/s00705-002-0852-0 12376753

[B44] Gavrilovskaya I. N. Shepley M. Shaw R. Ginsberg M. H. Mackow E. R. (1998). β_3_ integrins mediate the cellular entry of hantaviruses that cause respiratory failure. Proc. Natl. Acad. Sci. U.S.A. 95, 7074–7079. 10.1073/pnas.95.12.7074 9618541 PMC22743

[B45] Geimonen E. Neff S. Raymond T. Kocer S. S. Gavrilovskaya I. N. Mackow E. R. (2002). Pathogenic and nonpathogenic hantaviruses differentially regulate endothelial cell responses. Proc. Natl. Acad. Sci. U.S.A. 99, 13837–13842. 10.1073/pnas.192298899 12368479 PMC129784

[B46] Gizzi M. Delaere B. Weynand B. Clement J. Maes P. Vergote V.. (2013). Another case of “European hantavirus pulmonary syndrome” with severe lung, prior to kidney, involvement, and diagnosed by viral inclusions in lung macrophages. Eur. J. Clin. Microbiol. Infect. Dis. 32, 1341–1345. 10.1007/s10096-013-1885-x 23670277 PMC7102061

[B47] Glass G. E. Childs J. E. Korch G. W. LeDuc J. W. (1988). Association of intraspecific wounding with hantaviral infection in wild rats (*Rattus norvegicus*). Epidemiol. Infect. 101, 459–472. 10.1017/S0950268800054418 3141203 PMC2249393

[B48] Goeijenbier M. Hartskeerl R. A. Reimerink J. Verner-Carlsson J. Wagenaar J. F. Goris M. G.. (2014). The hanta hunting study: underdiagnosis of Puumala hantavirus infections in symptomatic non-travelling leptospirosis-suspected patients in the Netherlands, in 2010 and April to November 2011. Euro Surveill. 19:20878. 10.2807/1560-7917.ES2014.19.32.20878 25139076

[B49] Goeijenbier M. Meijers J. C. Anfasa F. Roose J. M. van de Weg C. A. Bakhtiari K.. (2015). Effect of Puumala hantavirus infection on human umbilical vein endothelial cell hemostatic function: platelet interactions, increased tissue factor expression and fibrinolysis regulator release. Front. Microbiol. 6:220. 10.3389/fmicb.2015.00220 25852676 PMC4371750

[B50] Golden J. W. Hammerbeck C. D. Mucker E. M. Brocato R. L. (2015). Animal models for the study of rodent-borne hemorrhagic fever viruses: arenaviruses and hantaviruses. Biomed. Res. Int. 2015:793257 10.1155/2015/793257 26266264 PMC4523679

[B51] Goldsmith C. S. Elliott L. H. Peters C. J. Zaki S. R. (1995). Ultrastructural characteristics of Sin Nombre virus, causative agent of hantavirus pulmonary syndrome. Arch. Virol. 140, 2107–2122. 10.1007/BF01323234 8572935

[B52] Gorbunova E. Gavrilovskaya I. N. Mackow E. R. (2010). Pathogenic hantaviruses Andes virus and Hantaan virus induce adherens junction disassembly by directing vascular endothelial cadherin internalization in human endothelial cells. J. Virol. 84, 7405–7411. 10.1128/JVI.00576-10 20463083 PMC2898267

[B53] Gorbunova E. E. Gavrilovskaya I. N. Pepini T. Mackow E. R. (2011). VEGFR2 and Src kinase inhibitors suppress Andes virus-induced endothelial cell permeability. J. Virol. 85, 2296–2303. 10.1128/JVI.02319-10 21177802 PMC3067787

[B54] Green W. Feddersen R. Yousef O. Behr M. Smith K. Nestler J.. (1998). Tissue distribution of hantavirus antigen in naturally infected humans and deer mice. J. Infect. Dis. 177, 1696–1700. 10.1086/515325 9607851

[B55] Groen J. Bruijn J. A. Gerding M. N. Jordans J. G. Moll van Charante A. W. Osterhaus A. D. (1996). Hantavirus antigen detection in kidney biopsies from patients with nephropathia epidemica. Clin. Nephrol. 46, 379–383. 8982553

[B56] Groen J. Gerding M. Koeman J. P. Roholl P. J. van Amerongen G. Jordans H. G.. (1995). A macaque model for hantavirus infection. J. Infect. Dis. 172, 38–44. 10.1093/infdis/172.1.38 7797944

[B57] Groeneveld P. H. Colson P. Kwappenberg K. M. Clement J. (1995). Increased production of nitric oxide in patients infected with the European variant of hantavirus. Scand. J. Infect. Dis. 27, 453–456. 10.3109/00365549509047045 8588134

[B58] Hagele S. Muller A. Nusshag C. Reiser J. Zeier M. Krautkramer E. (2018). Motility of human renal cells is disturbed by infection with pathogenic hantaviruses. BMC Infect. Dis. 18:645. 10.1186/s12879-018-3583-x 30541481 PMC6292036

[B59] Hagele S. Muller A. Nusshag C. Reiser J. Zeier M. Krautkramer E. (2019). Virus- and cell type-specific effects in orthohantavirus infection. Virus Res. 260, 102–113. 10.1016/j.virusres.2018.11.015 30508604

[B60] Hallin G. W. Simpson S. Q. Crowell R. E. James D. S. Koster F. T. Mertz G. J.. (1996). Cardiopulmonary manifestations of hantavirus pulmonary syndrome. Crit. Care Med. 24, 252–258. 10.1097/00003246-199602000-00012 8605797

[B61] Hammerbeck C. D. Hooper J. W. (2011). T cells are not required for pathogenesis in the Syrian hamster model of hantavirus pulmonary syndrome. J. Virol. 85, 9929–9944. 10.1128/JVI.05356-11 21775442 PMC3196444

[B62] Hardestam J. Karlsson M. Falk K. I. Olsson G. Klingstrom J. Lundkvist A. (2008). Puumala hantavirus excretion kinetics in bank voles (*Myodes glareolus*). Emerg. Infect. Dis. 14, 1209–1215. 10.3201/eid1408.080221 18680643 PMC2600398

[B63] Hautala T. Sironen T. Vapalahti O. Paakko E. Sarkioja T. Salmela P. I.. (2002). Hypophyseal hemorrhage and panhypopituitarism during Puumala virus infection: magnetic resonance imaging and detection of viral antigen in the hypophysis. Clin. Infect. Dis. 35, 96–101. 10.1086/340859 12060884

[B64] Hepojoki J. Vaheri A. Strandin T. (2014). The fundamental role of endothelial cells in hantavirus pathogenesis. Front. Microbiol. 5:727. 10.3389/fmicb.2014.00727 25566236 PMC4273638

[B65] Hjelle B. (2002). Vaccines against hantaviruses. Expert Rev. Vaccines 1, 373–384. 10.1586/14760584.1.3.373 12901576

[B66] Hjelle B. Torres-Perez F. (2010). Hantaviruses in the americas and their role as emerging pathogens. Viruses 2, 2559–2586. 10.3390/v2122559 21994631 PMC3185593

[B67] Hooper J. W. Larsen T. Custer D. M. Schmaljohn C. S. (2001). A lethal disease model for hantavirus pulmonary syndrome. Virology 289, 6–14. 10.1006/viro.2001.1133 11601912

[B68] Hung T. Zhou J. Y. Tang Y. M. Zhao T. X. Baek L. J. Lee H. W. (1992). Identification of Hantaan virus-related structures in kidneys of cadavers with haemorrhagic fever with renal syndrome. Arch. Virol. 122, 187–199. 10.1007/BF01321127 1346088

[B69] Jangra R. K. Herbert A. S. Li R. Jae L. T. Kleinfelter L. M. Slough M. M.. (2018). Protocadherin-1 is essential for cell entry by new world hantaviruses. Nature 563, 559–563. 10.1038/s41586-018-0702-1 30464266 PMC6556216

[B70] Jiang H. Zheng X. Wang L. Du H. Wang P. Bai X. (2017). Hantavirus infection: a global zoonotic challenge. Virol. Sin. 32, 32–43. 10.1007/s12250-016-3899-x 28120221 PMC6598904

[B71] Jonsson C. B. Figueiredo L. T. Vapalahti O. (2010). A global perspective on hantavirus ecology, epidemiology, and disease. Clin. Microbiol. Rev. 23, 412–441. 10.1128/CMR.00062-09 20375360 PMC2863364

[B72] Kariwa H. Fujiki M. Yoshimatsu K. Arikawa J. Takashima I. Hashimoto N. (1998). Urine-associated horizontal transmission of Seoul virus among rats. Arch. Virol. 143, 365–374. 10.1007/s007050050292 9541619

[B73] Khaiboullina S. F. Levis S. Morzunov S. P. Martynova E. V. Anokhin V. A. Gusev O. A.. (2017). Serum cytokine profiles differentiating hemorrhagic fever with renal syndrome and hantavirus pulmonary syndrome. Front. Immunol. 8:567. 10.3389/fimmu.2017.00567 28572804 PMC5435745

[B74] Khan A. S. Ksiazek T. G. Peters C. J. (1996). Hantavirus pulmonary syndrome. Lancet 347, 739–741. 10.1016/S0140-6736(96)90082-3 8602007

[B75] Kim S. Kang E. T. Kim Y. G. Han J. S. Lee J. S. Kim Y. I.. (1993). Localization of Hantaan viral envelope glycoproteins by monoclonal antibodies in renal tissues from patients with Korean hemorrhagic fever H. Am. J. Clin. Pathol. 100, 398–403. 10.1093/ajcp/100.4.398 7692720

[B76] Kim Y. S. Ahn C. Han J. S. Kim S. Lee J. S. Lee P. W. (1995). Hemorrhagic fever with renal syndrome caused by the Seoul virus. Nephron 71, 419–427. 10.1159/000188762 8587622

[B77] Kitterer D. Greulich S. Grun S. Segerer S. Mustonen J. Alscher M. D.. (2016). Electrocardiographic abnormalities and relative bradycardia in patients with hantavirus-induced nephropathia epidemica. Eur. J. Intern. Med. 33, 67–73. 10.1016/j.ejim.2016.06.001 27296590

[B78] Klein S. L. Bird B. H. Glass G. E. (2001). Sex differences in immune responses and viral shedding following Seoul virus infection in Norway rats. Am. J. Trop. Med. Hyg. 65, 57–63. 10.4269/ajtmh.2001.65.57 11504409

[B79] Klingstrom J. Plyusnin A. Vaheri A. Lundkvist A. (2002). Wild-type Puumala hantavirus infection induces cytokines, C-reactive protein, creatinine, and nitric oxide in cynomolgus macaques. J. Virol. 76, 444–449. 10.1128/JVI.76.1.444-449.2002 11739712 PMC135710

[B80] Koskela S. M. Laine O. K. Paakkala A. S. Makela S. M. Mustonen J. T. (2014). Spleen enlargement is a common finding in acute Puumala hantavirus infection and it does not associate with thrombocytopenia. Scand. J. Infect. Dis. 46, 723–726. 10.3109/00365548.2014.930967 25119440

[B81] Kottke M. A. Walters T. J. (2016). Where's the leak in vascular barriers? A review. Shock 46, 20–36. 10.1097/SHK.0000000000000666 27405062

[B82] Kraus A. A. Raftery M. J. Giese T. Ulrich R. Zawatzky R. Hippenstiel S.. (2004). Differential antiviral response of endothelial cells after infection with pathogenic and nonpathogenic hantaviruses. J. Virol. 78, 6143–6150. 10.1128/JVI.78.12.6143-6150.2004 15163707 PMC416501

[B83] Krautkramer E. Grouls S. Hettwer D. Rafat N. Tonshoff B. Zeier M. (2014). Mobilization of circulating endothelial progenitor cells correlates with the clinical course of hantavirus disease. J. Virol. 88, 483–489. 10.1128/JVI.02063-13 24155401 PMC3911717

[B84] Krautkramer E. Grouls S. Stein N. Reiser J. Zeier M. (2011). Pathogenic old world hantaviruses infect renal glomerular and tubular cells and induce disassembling of cell-to-cell contacts. J. Virol. 85, 9811–9823. 10.1128/JVI.00568-11 21775443 PMC3196447

[B85] Krautkramer E. Zeier M. (2008). Hantavirus causing hemorrhagic fever with renal syndrome enters from the apical surface and requires decay-accelerating factor (DAF/CD55). J. Virol. 82, 4257–4264. 10.1128/JVI.02210-07 18305044 PMC2293039

[B86] Krautkramer E. Zeier M. Plyusnin A. (2013). Hantavirus infection: an emerging infectious disease causing acute renal failure. Kidney Int. 83, 23–27. 10.1038/ki.2012.360 23151954

[B87] Kuiken T. Taubenberger J. K. (2008). Pathology of human influenza revisited. Vaccine 26, 59–66. 10.1016/j.vaccine.2008.07.025 19230162 PMC2605683

[B88] Larson R. S. Brown D. C. Ye C. Hjelle B. (2005). Peptide antagonists that inhibit Sin Nombre virus and hantaan virus entry through the beta3-integrin receptor. J. Virol. 79, 7319–7326. 10.1128/JVI.79.12.7319-7326.2005 15919886 PMC1143646

[B89] Latus J. Kitterer D. Segerer S. Artunc F. Alscher M. D. Braun N. (2015). Severe thrombocytopenia in hantavirus-induced nephropathia epidemica. Infection 43, 83–87. 10.1007/s15010-014-0699-9 25380569

[B90] Latus J. Tenner-Racz K. Racz P. Kitterer D. Cadar D. Ott G.. (2014). Detection of Puumala hantavirus antigen in human intestine during acute hantavirus infection. PLoS ONE 9:e98397. 10.1371/journal.pone.0098397 24857988 PMC4032337

[B91] LeDuc J. W. Smith G. A. Johnson K. M. (1984). Hantaan-like viruses from domestic rats captured in the United States. Am. J. Trop. Med. Hyg. 33, 992–998. 10.4269/ajtmh.1984.33.992 6435464

[B92] Lee H. W. Baek L. J. Johnson K. M. (1982). Isolation of Hantaan virus, the etiologic agent of Korean hemorrhagic fever, from wild urban rats. J. Infect. Dis. 146, 638–644. 10.1093/infdis/146.5.638 6127366

[B93] Lee H. W. Johnson K. M. (1982). Laboratory-acquired infections with Hantaan virus, the etiologic agent of Korean hemorrhagic fever. J. Infect. Dis. 146, 645–651. 10.1093/infdis/146.5.645 6127367

[B94] Lee H. W. Lee P. W. Baek L. J. Song C. K. Seong I. W. (1981). Intraspecific transmission of Hantaan virus, etiologic agent of Korean hemorrhagic fever, in the rodent *Apodemus agrarius*. Am. J. Trop. Med. Hyg. 30, 1106–1112. 10.4269/ajtmh.1981.30.1106 6116436

[B95] Lee M. (1987). Coagulopathy in patients with hemorrhagic fever with renal syndrome. J. Korean Med. Sci. 2, 201–211. 10.3346/jkms.1987.2.4.201 3151765 PMC3053648

[B96] Li W. Klein S. L. (2012). Seoul virus-infected rat lung endothelial cells and alveolar macrophages differ in their ability to support virus replication and induce regulatory T cell phenotypes. J. Virol. 86, 11845–11855. 10.1128/JVI.01233-12 22915818 PMC3486303

[B97] Liu L. B. Xue Y. X. Liu Y. H. Wang Y. B. (2008). Bradykinin increases blood-tumor barrier permeability by down-regulating the expression levels of ZO-1, occludin, and claudin-5 and rearranging actin cytoskeleton. J. Neurosci. Res. 86, 1153–1168. 10.1002/jnr.21558 18183615

[B98] Lutteke N. Raftery M. J. Lalwani P. Lee M. H. Giese T. Voigt S.. (2010). Switch to high-level virus replication and HLA class I upregulation in differentiating megakaryocytic cells after infection with pathogenic hantavirus. Virology 405, 70–80. 10.1016/j.virol.2010.05.028 20673746

[B99] Maas M. van Heteren M. de Vries A. Kuiken T. Hoornweg T. Veldhuis Kroeze E.. (2019). Seoul virus tropism and pathology in naturally infected feeder rats. Viruses 11:531. 10.3390/v11060531 31181690 PMC6630879

[B100] Mackow E. R. Gorbunova E. E. Gavrilovskaya I. N. (2014). Endothelial cell dysfunction in viral hemorrhage and edema. Front. Microbiol. 5:733. 10.3389/fmicb.2014.00733 25601858 PMC4283606

[B101] Macneil A. Nichol S. T. Spiropoulou C. F. (2011). Hantavirus pulmonary syndrome. Virus Res. 162, 138–147. 10.1016/j.virusres.2011.09.017 21945215

[B102] Maes P. Clement J. Groeneveld P. H. Colson P. Huizinga T. W. Van Ranst M. (2006). Tumor necrosis factor-alpha genetic predisposing factors can influence clinical severity in nephropathia epidemica. Viral Immunol. 19, 558–564. 10.1089/vim.2006.19.558 16987073

[B103] Martinez-Valdebenito C. Calvo M. Vial C. Mansilla R. Marco C. Palma R. E.. (2014). Person-to-person household and nosocomial transmission of andes hantavirus, Southern Chile, 2011. Emerg. Infect. Dis. 20, 1629–1636. 10.3201/eid2010.140353 25272189 PMC4193174

[B104] Meier M. Kramer J. Jabs W. J. Nolte C. Hofmann J. Kruger D. H.. (2018). Proteinuria and the clinical course of Dobrava-Belgrade hantavirus infection. Nephron Extra 8, 1–10. 10.1159/000486322 29849535 PMC5968261

[B105] Michalski A. Niemcewicz M. Bielawska-Drozd A. Nowakowska A. Gawel J. Pitucha G.. (2014). Surveillance of hantaviruses in Poland: a study of animal reservoirs and human hantavirus disease in Subcarpathia. Vector Borne Zoonotic Dis. 14, 514–522. 10.1089/vbz.2013.1468 24902039 PMC4098853

[B106] Mori M. Rothman A. L. Kurane I. Montoya J. M. Nolte K. B. Norman J. E.. (1999). High levels of cytokine-producing cells in the lung tissues of patients with fatal hantavirus pulmonary syndrome. J. Infect. Dis. 179, 295–302. 10.1086/314597 9878011

[B107] Mustonen J. Outinen T. Laine O. Porsti I. Vaheri A. Makela S. (2017). Kidney disease in Puumala hantavirus infection. Infect. Dis. 49, 321–332. 10.1080/23744235.2016.1274421 28049381

[B108] Netski D. Thran B. H. St Jeor S. C. (1999). Sin Nombre virus pathogenesis in Peromyscus maniculatus. J. Virol. 73, 585–591. 10.1128/JVI.73.1.585-591.1999 9847363 PMC103864

[B109] Nielsen C. F. Sethi V. Petroll A. E. Kazmierczak J. Erickson B. R. Nichol S. T.. (2010). Seoul virus infection in a Wisconsin patient with recent travel to China, March 2009: first documented case in the Midwestern United States. Am. J. Trop. Med. Hyg. 83, 1266–1268 10.4269/ajtmh.2010.10-0424 21118933 PMC2990043

[B110] Nolte K. B. Feddersen R. M. Foucar K. Zaki S. R. Koster F. T. Madar D.. (1995). Hantavirus pulmonary syndrome in the United States: a pathological description of a disease caused by a new agent. Hum. Pathol. 26, 110–120. 10.1016/0046-8177(95)90123-X 7821907

[B111] Padula P. Figueroa R. Navarrete M. Pizarro E. Cadiz R. Bellomo C.. (2004). Transmission study of Andes hantavirus infection in wild sigmodontine rodents. J. Virol. 78, 11972–11979. 10.1128/JVI.78.21.11972-11979.2004 15479837 PMC523238

[B112] Padula P. J. Edelstein A. Miguel S. D. Lopez N. M. Rossi C. M. Rabinovich R. D. (1998). Hantavirus pulmonary syndrome outbreak in Argentina: molecular evidence for person-to-person transmission of Andes virus. Virology 241, 323–330. 10.1006/viro.1997.8976 9499807

[B113] Papa A. (2012). Dobrava-Belgrade virus: phylogeny, epidemiology, disease. Antiviral Res. 95, 104–117. 10.1016/j.antiviral.2012.05.011 22659378

[B114] Passaro D. J. Shieh W. J. Hacker J. K. Fritz C. L. Hogan S. R. Fischer M.. (2001). Predominant kidney involvement in a fatal case of hantavirus pulmonary syndrome caused by Sin Nombre virus. Clin. Infect. Dis. 33, 263–264. 10.1086/321832 11418889

[B115] Peters C. J. Khan A. S. (2002). Hantavirus pulmonary syndrome: the new American hemorrhagic fever. Clin. Infect. Dis. 34, 1224–1231. 10.1086/339864 11941549

[B116] Peters C. J. Simpson G. L. Levy H. (1999). Spectrum of hantavirus infection: hemorrhagic fever with renal syndrome and hantavirus pulmonary syndrome. Annu. Rev. Med. 50, 531–545. 10.1146/annurev.med.50.1.531 10073292

[B117] Plyusnin A. Morzunov S. P. (2001). Virus evolution and genetic diversity of hantaviruses and their rodent hosts. Curr. Top. Microbiol. Immunol. 256, 47–75. 10.1007/978-3-642-56753-7_4 11217406

[B118] Puljiz I. Kuzman I. Markotic A. Turcinov D. Matic M. Makek N. (2005). Electrocardiographic changes in patients with haemorrhagic fever with renal syndrome. Scand. J. Infect. Dis. 37, 594–598. 10.1080/00365540510036606 16138429

[B119] Rasmuson J. Andersson C. Norrman E. Haney M. Evander M. Ahlm C. (2011). Time to revise the paradigm of hantavirus syndromes? Hantavirus pulmonary syndrome caused by European hantavirus. Eur. J. Clin. Microbiol. Infect. Dis. 30, 685–690. 10.1007/s10096-010-1141-6 21234633 PMC3075397

[B120] Rasmuson J. Lindqvist P. Sorensen K. Hedstrom M. Blomberg A. Ahlm C. (2013). Cardiopulmonary involvement in Puumala hantavirus infection. BMC Infect. Dis. 13:501. 10.1186/1471-2334-13-501 24160911 PMC4231367

[B121] Rasmuson J. Pourazar J. Mohamed N. Lejon K. Evander M. Blomberg A.. (2016). Cytotoxic immune responses in the lungs correlate to disease severity in patients with hantavirus infection. Eur. J. Clin. Microbiol. Infect. Dis. 35, 713–721. 10.1007/s10096-016-2592-1 26873376 PMC4819462

[B122] Reusken C. Heyman P. (2013). Factors driving hantavirus emergence in Europe. Curr. Opin. Virol. 3, 92–99. 10.1016/j.coviro.2013.01.002 23384818

[B123] Robinson S. D. Reynolds L. E. Wyder L. Hicklin D. J. Hodivala-Dilke K. M. (2004). Beta3-integrin regulates vascular endothelial growth factor-A-dependent permeability. Arterioscler. Thromb. Vasc. Biol. 24, 2108–2114. 10.1161/01.ATV.0000143857.27408.de 15345507

[B124] Rowe R. K. Pekosz A. (2006). Bidirectional virus secretion and nonciliated cell tropism following Andes virus infection of primary airway epithelial cell cultures. J. Virol. 80, 1087–1097. 10.1128/JVI.80.3.1087-1097.2006 16414986 PMC1346943

[B125] Sabatier F. Camoin-Jau L. Anfosso F. Sampol J. Dignat-George F. (2009). Circulating endothelial cells, microparticles and progenitors: key players towards the definition of vascular competence. J. Cell. Mol. Med. 13, 454–471. 10.1111/j.1582-4934.2008.00639.x 19379144 PMC3822508

[B126] Safronetz D. Zivcec M. Lacasse R. Feldmann F. Rosenke R. Long D.. (2011). Pathogenesis and host response in Syrian hamsters following intranasal infection with Andes virus. PLoS Pathog. 7:e1002426. 10.1371/journal.ppat.1002426 22194683 PMC3240607

[B127] Saggioro F. P. Rossi M. A. Duarte M. I. Martin C. C. Alves V. A. Moreli M. L.. (2007). Hantavirus infection induces a typical myocarditis that may be responsible for myocardial depression and shock in hantavirus pulmonary syndrome. J. Infect. Dis. 195, 1541–1549. 10.1086/513874 17436235

[B128] Sane J. Reimerink J. Harms M. Bakker J. Mughini-Gras L. Schimmer B.. (2014). Human hantavirus infections in the Netherlands. Emerg. Infect. Dis. 20, 2107–2110. 10.3201/eid2012.131886 25417752 PMC4257821

[B129] Schmaljohn C. Hjelle B. (1997). Hantaviruses: a global disease problem. Emerg. Infect. Dis. 3, 95–104. 10.3201/eid0302.970202 9204290 PMC2627612

[B130] Schountz T. Prescott J. Cogswell A. C. Oko L. Mirowsky-Garcia K. Galvez A. P.. (2007). Regulatory T cell-like responses in deer mice persistently infected with Sin Nombre virus. Proc. Natl. Acad. Sci. U.S.A. 104, 15496–15501. 10.1073/pnas.0707454104 17875986 PMC2000535

[B131] Schutt M. Meisel H. Kruger D. H. Ulrich R. Dalhoff K. Dodt C. (2004). Life-threatening Dobrava hantavirus infection with unusually extended pulmonary involvement. Clin. Nephrol. 62, 54–57. 10.5414/CNP62054 15267014

[B132] Singh B. K. Li N. Mark A. C. Mateo M. Cattaneo R. Sinn P. L. (2016). Cell-to-cell contact and nectin-4 govern spread of Measles virus from primary human myeloid cells to primary human airway epithelial cells. J. Virol. 90, 6808–6817. 10.1128/JVI.00266-16 27194761 PMC4944272

[B133] Sironen T. Klingstrom J. Vaheri A. Andersson L. C. Lundkvist A. Plyusnin A. (2008). Pathology of Puumala hantavirus infection in macaques. PLoS ONE 3:e3035. 10.1371/journal.pone.0003035 18716663 PMC2516326

[B134] Sironen T. Sane J. Lokki M. L. Meri S. Andersson L. C. Hautala T.. (2017). Fatal Puumala hantavirus disease: involvement of complement activation and vascular leakage in the pathobiology. Open Forum Infect. Dis. 4:ofx229. 10.1093/ofid/ofx229 29255728 PMC5726462

[B135] Somanath P. R. Malinin N. L. Byzova T. V. (2009). Cooperation between integrin alphavbeta3 and VEGFR2 in angiogenesis. Angiogenesis 12, 177–185. 10.1007/s10456-009-9141-9 19267251 PMC2863048

[B136] Suh D. C. Park J. S. Park S. K. Lee H. K. Chang K. H. (1995). Pituitary hemorrhage as a complication of hantaviral disease. AJNR Am. J. Neuroradiol. 16, 175–178; discussion 179–180. 7900589 PMC8337687

[B137] Swanink C. Reimerink J. Gisolf J. de Vries A. Claassen M. Martens L.. (2018). Autochthonous human case of Seoul virus infection, the Netherlands. Emerg. Infect. Dis. 24, 2158–2163. 10.3201/eid2412.180229 30067176 PMC6256391

[B138] Swerlick R. A. Lawley T. J. (1993). Role of microvascular endothelial cells in inflammation. J. Invest. Dermatol. 100, 111–115. 10.1038/jid.1993.33 8423379

[B139] Taylor S. L. Wahl-Jensen V. Copeland A. M. Jahrling P. B. Schmaljohn C. S. (2013). Endothelial cell permeability during hantavirus infection involves factor XII-dependent increased activation of the kallikrein-kinin system. PLoS Pathog. 9:e1003470. 10.1371/journal.ppat.1003470 23874198 PMC3715459

[B140] Temonen M. Mustonen J. Helin H. Pasternack A. Vaheri A. Holthofer H. (1996). Cytokines, adhesion molecules, and cellular infiltration in nephropathia epidemica kidneys: an immunohistochemical study. Clin. Immunol. Immunopathol. 78, 47–55. 10.1006/clin.1996.0007 8599883

[B141] Teoh C. M. Tan S. S. L. Tran T. (2016). Integrins as therapeutic targets for respiratory diseases. Curr. Mol. Med. 15, 714–734. 10.2174/1566524015666150921105339 26391549 PMC5427774

[B142] Terajima M. Ennis F. A. (2011). T cells and pathogenesis of hantavirus cardiopulmonary syndrome and hemorrhagic fever with renal syndrome. Viruses 3, 1059–1073. 10.3390/v3071059 21994770 PMC3185782

[B143] Tkachenko E. A. Ishmukhametov A. A. Dzagurova T. K. Bernshtein A. D. Morozov V. G. Siniugina A. A.. (2019). Hemorrhagic fever with renal syndrome, Russia. Emerg. Infect. Dis. 25, 2325–2328. 10.3201/eid2512.181649 31742540 PMC6874259

[B144] Toro J. Vega J. D. Khan A. S. Mills J. N. Padula P. Terry W.. (1998). An outbreak of hantavirus pulmonary syndrome, Chile, 1997. Emerg. Infect. Dis. 4, 687–694. 10.3201/eid0404.980425 9866751 PMC2640255

[B145] Vaheri A. Strandin T. Hepojoki J. Sironen T. Henttonen H. Makela S.. (2013). Uncovering the mysteries of hantavirus infections. Nat. Rev. Microbiol. 11, 539–550. 10.1038/nrmicro3066 24020072

[B146] van Riel D. den Bakker M. A. Leijten L. M. Chutinimitkul S. Munster V. J. de Wit E.. (2010). Seasonal and pandemic human influenza viruses attach better to human upper respiratory tract epithelium than avian influenza viruses. Am. J. Pathol. 176, 1614–1618. 10.2353/ajpath.2010.090949 20167867 PMC2843453

[B147] Voutilainen L. Sironen T. Tonteri E. Back A. T. Razzauti M. Karlsson M.. (2015). Life-long shedding of Puumala hantavirus in wild bank voles (*Myodes glareolus*). J. Gen. Virol. 96, 1238–1247. 10.1099/vir.0.000076 25701819

[B148] Wahl-Jensen V. Chapman J. Asher L. Fisher R. Zimmerman M. Larsen T.. (2007). Temporal analysis of Andes virus and Sin Nombre virus infections of Syrian hamsters. J. Virol. 81, 7449–7462. 10.1128/JVI.00238-07 17475651 PMC1933362

[B149] Xiao R. Yang S. Koster F. Ye C. Stidley C. Hjelle B. (2006). Sin Nombre viral RNA load in patients with hantavirus cardiopulmonary syndrome. J. Infect. Dis. 194, 1403–1409. 10.1086/508494 17054070

[B150] Yanagihara R. Amyx H. L. Gajdusek D. C. (1985). Experimental infection with Puumala virus, the etiologic agent of nephropathia epidemica, in bank voles (*Clethrionomys glareolus*). J. Virol. 55, 34–38. 10.1128/JVI.55.1.34-38.1985 2861296 PMC254894

[B151] Yanagihara R. Silverman D. J. (1990). Experimental infection of human vascular endothelial cells by pathogenic and nonpathogenic hantaviruses. Arch. Virol. 111, 281–286. 10.1007/BF01311063 2112908

[B152] Zaki S. R. Greer P. W. Coffield L. M. Goldsmith C. S. Nolte K. B. Foucar K.. (1995). Hantavirus pulmonary syndrome. Pathogenesis of an emerging infectious disease. Am. J. Pathol. 146, 552–579. 7887439 PMC1869168

[B153] Zelena H. Mrazek J. Kuhn T. (2013). Tula hantavirus infection in immunocompromised host, Czech Republic. Emerg. Infect. Dis. 19, 1873–1875. 10.3201/eid1911.130421 24209605 PMC3837639

[B154] Zhang X. Chen H. Y. Zhu L. Y. Zeng L. L. Wang F. Li Q. G.. (2011). Comparison of Hantaan and Seoul viral infections among patients with hemorrhagic fever with renal syndrome (HFRS) in Heilongjiang, China. Scand. J. Infect. Dis. 43, 632–641. 10.3109/00365548.2011.566279 21428852

[B155] Zhang Y. Z. (2014). Discovery of hantaviruses in bats and insectivores and the evolution of the genus Hantavirus. Virus Res. 187, 15–21. 10.1016/j.virusres.2013.12.035 24509342

